# Haematemesis due to primary aortic aneurysm-duodenal fistula - clinical suspicion is the cornerstone of diagnosis: a case report

**DOI:** 10.4076/1757-1626-2-7803

**Published:** 2009-06-09

**Authors:** Serosha Mandika Wijeyaratne, Ranjuka Ubayasiri, Charitha Weerasinghe

**Affiliations:** Department of Surgery, Faculty of MedicineKynsey Road, Colombo 8Sri Lanka

## Abstract

**Introduction:**

Although gastrointestinal haemorrhage from aortoduodenal fistulae secondary to previous aortic grafts are well known, a primary fistula from an aortic aneurysm is a rare consideration resulting in inappropriate management and poor outcomes.

**Case presentation:**

We report a previously fit 65-year-old Sri Lankan man who presented with severe anaemia (haemoglobin, 6 gm/dl), recent onset low backache. There was no history of analgesic abuse, peptic ulceration, alcohol excess, weight loss or malena. The abdomen was soft and there was no visceromegaly. A routine ultrasound detected an abdominal aortic aneurysm without signs of a leak. Two days later, while undergoing routine diagnostic tests for anaemia and backache, he had a massive haematemesis. Standard resuscitation was commenced with hope that common sources, either peptic ulcers or varicies would eventually stop bleeding enabling endoscopy and definitive treatment. However, persistent hypotension coupled with the clinical suspicion of an aortoduodenal fistula led to immediate surgical exploration rather than continued aggressive resuscitation. An aortoduodenal fistula was confirmed and both the duodenum and the aorta were successfully repaired by direct suture and synthetic graft replacement respectively. This man remains well nine months later.

**Conclusion:**

Gastrointestinal bleeding in the presence of an ‘asymptomatic’ abdominal aortic aneurysms should be assumed to be from a primary aortoduodenal fistula unless another source can be identified with certainty without delay.

## Introduction

Abdominal aortic aneurysms (AAA) and haematemesis are common clinical problems which are expected to increase as the population ages. An AAA is usually detected either incidentally on routine palpation and ultrasound examinations or when complicated with pain or extraperitoneal rupture. Haematemesis is commonly from gastro-oesophageal varicies or ulceration. Nevertheless, rupture of an AAA in to the duodenum, an aortoduodenal fistula (ADF) is a rare complication of an AAA [1] and haematemesis is rarely caused by ADF. This explains diagnostic and therapeutic delays with primary ADF and resultant high mortality.

## Case presentation

**Figure 1.**
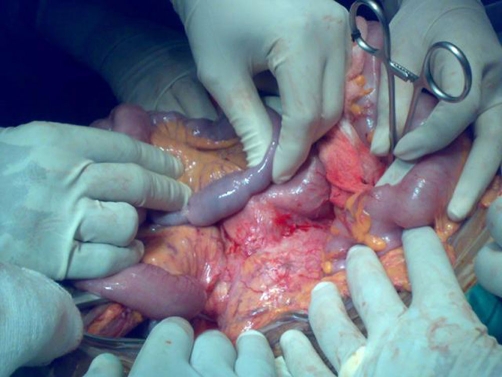
Intraoperative view from the left side of the patient showing the adherence of the third part of duodenum to the lower right lateral wall of the aneurysm. The loop of intestine held between fingers shows blood within.

**Figure 2.**
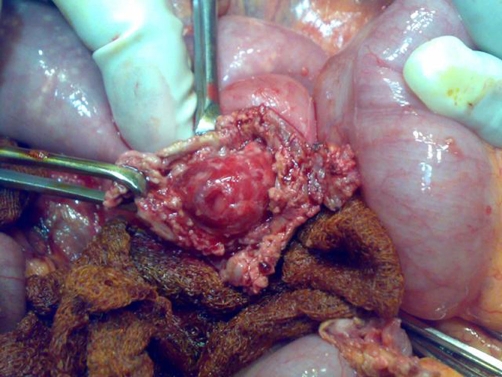
Intraoperative view of the opening in the third part of duodenum surrounded by adherent aneurysm wall held with an instrument.

We report a 65-year-old Sri Lankan man who was hospitalised with recent onset low backache and severe pallor (haemoglobin, 6 gm/dl). He had been previously well and there was no history of ingestion of aspirin or non-steroidal anti-inflammatory drugs, alcohol abuse, chronic abdominal pain, weight loss, change in bowel habits or malena. He had given up tobacco smoking five years back. He was not on anticoagulants. His abdomen was soft and there was no visceromegaly. Investigations were being arranged to determine a cause for his anaemia and backache and a routine abdominal ultrasound detected a 6 × 7 cm abdominal aortic aneurysm with no signs of a leak. On the second day after hospitalisation he had a massive haematemesis and became hypotensive (systolic blood pressure 70 mm Hg). The emergency care team commenced resuscitation with blood and considered common causes such as gastro-oesophageal varicies and ulcers. However, the poor initial response to fluids and clinical suspicion of an ADF led us to hold back continued aggressive resuscitation until the source was controlled at surgery. At laparotomy gastro-oesophageal varicies and chronic ulceration were ruled out. The third part of the duodenum was densely adherent to the right lower lateral wall of the aneurysm and there was no evidence of overt sepsis (Figure 1.). The aneurysm was opened into after clamping the aorta. The fistulous connection was visualized from within (Figure 2.) after evacuation of clots. The AAA was repaired with a 16 mm diameter Polytetrafluoroethylene Gore-Tex^®^ tube graft, and the duodenum was closed in two layers. An omentopexy was performed between the aorta-graft anastomosis and duodenum. The patient made an uneventful recovery. Although intraoperative bacterial cultures were negative antimicrobial prophylaxis with co-trimoxazole was prescribed for two weeks. Nine months later, he remains well with a haemoglobin level of 12.5 gm/dl.

## Discussion

The need to consider an ADF as the cause of upper gastrointestinal bleeding in a patient with a previous aortic anastomosis is well established [2]. Interestingly, there are recent reports of ADF even after endovascular stent grafts for AAAs [3]. The thinking is not the same as when there is gastrointestinal bleeding in a patient with an incidental AAA. Furthermore, in primary ADF, the combination of a pulsatile abdominal mass and gastrointestinal bleeding is seen only in 23% [4] although this would increase with the use of ultrasound. But even when haematemesis occurs in a patient known to have an AAA initial management is limited to the more common causes such as varices and peptic ulceration. Thus in a hypotensive patient, blood is transfused until pressures are normalized and the bleeding usually stops spontaneously. In the case of an aortic bleed this would cause further bleeding and death. In a normotensive patient, upper gastrointestinal endoscopy would be the initial step in the search for a cause. However, finding lesions without active bleeding does not rule out an ADF and the length of the endoscope does not allow visualization of the distal duodenum where ADF occur [5]. Computed tomography with contrast is probably the most useful as it may show the communication or reveal loss of continuity and air bubbles in the aneurysm wall that are pathognomonic [6]. Percutaneous angiography is rarely of value since the need for it coincides with the need for immediate surgery [7].

The pattern of bleeding from an ADF is of interest. A “herald” haemorrhage, which may be occult or mild, is followed hours, days, or weeks later by catastrophic haemorrhage that is characteristic of an ADF. This initial slow bleed is the result of a small fistula occluded by thrombus. Since 70% survive at least 6 hours [4] and up to 50%, 24 hours [8] after the initial bleed, a “herald” haemorrhage should be viewed as an opportunity for prompt surgical intervention. Despite technological advances in endoscopy and imaging, the cornerstone in the diagnosis of an ADF remains clinical suspicion. The challenge is even greater when the initial bleed is occult and manifest only as anaemia as in our patient. In this instance, the non tender AAA with negative imaging for a leak was not considered the source of bleeding and a primary haematological or gastrointestinal cause was being pursued.

Surgical repair without delay is the only chance for survival [4]. Surgery must disconnect the fistula, debride the retroperitoneum, repair the duodenum and aorta with omental interposition and establish blood flow to the lower extremities. In the absence of gross infection as in our patient, anatomic in situ repair of the aorta with a synthetic graft gives good results [9]. However, when there is gross infection as with mycotic aneurysms, closure of the aorta and extra-anatomic grafting is advisable [10]. More recently, in situ grafting using autogenous vein, either superficial femoral vein [11] or spiraled saphenous vein [12] has been recommended. Finally, successful endovascular stent grafting has been reported in the case of a very high risk patient giving us an additional option for the future [13].

## Conclusion

Even though primary ADF is a rare cause of enteric bleeding, what makes it important is the high mortality from lack of awareness and non diagnosis. The key to its successful management is a high degree of clinical suspicion when there is gastrointestinal bleeding in combination with an AAA. We recommend that in such instances bleeding must be assumed to be from an ADF unless another source can be identified with certainty. The search for other causes is advisable only in normotensive patients while hypotensive patients should be referred to vascular surgeons to be operated on without further delay.

## List of abbreviations

AAA: Abdominal aortic aneurysms
ADF: Aortoduodenal fistula
